# Systemic Immune-Inflammation Index and Related Hematologic Markers as Prognostic Tools in Type 2 Diabetes

**DOI:** 10.3390/medicina61081433

**Published:** 2025-08-09

**Authors:** Raluca Cosma-Lăzuran, Daniel-Corneliu Leucuta, Mihaela-Simona Popoviciu

**Affiliations:** 1 Faculty of Medicine and Pharmacy, University of Oradea, Piața 1 Decembrie Street nr 10, 410073 Oradea, Romania; lazuran.raluca@student.uoradea.ro; 2 Department of Medical Informatics and Biostatistics, “Iuliu Hatieganu” University of Medicine and Pharmacy, 400349 Cluj-Napoca, Romania; 3 Departament of Diabetes, Nutrition and Metabolic Diseases-Clinical Section Internal Medicine I, Bihor County Emergency Clinical Hospital, 410087 Oradea, Romania; 4 Departament of Clinical Disciplines, Faculty of Medicine and Pharmacy, University of Oradea, Piața 1 Decembrie Street nr 10, 410073 Oradea, Romania

**Keywords:** type 2 diabetes mellitus, inflammatory markers, neutrophil-to-lymphocyte ratio, systemic immune-inflammation index, systemic inflammation response index, aggregate index of systemic inflammation

## Abstract

*Background and Objectives:* Chronic low-grade inflammation plays a key role in the pathogenesis of type 2 diabetes mellitus (T2DM) and its vascular complications. Hematological indices derived from routine blood counts, such as neutrophil-to-lymphocyte ratio (NLR), derived neutrophil-to-lymphocyte ratio (dNLR), platelet-to-lymphocyte ratio (PLR), lymphocyte-to-monocyte ratio (LMR), systemic immune-inflammation index (SII), systemic inflammation response index (SIRI), and aggregate index of systemic inflammation (AISI), have been proposed as surrogate markers for systemic inflammation and predictors of cardiovascular risk. This study aimed to evaluate the predictive value of these inflammatory indices concerning the presence of micro- and macrovascular complications and cardiovascular mortality in patients with type 2 diabetes mellitus. *Materials and Methods:* We conducted a retrospective cohort study including 237 patients with T2DM. We assessed the association between hematological indices and cardiovascular mortality, followed by a ROC curve analysis to evaluate their predictive performance, and a multiple logistic regression. *Results:* Thirty patients (12.66%) died during the study period. ROC analysis showed that SIRI (AUC = 0.680 [95% CI 0.576–0.779]), LMR (AUC = 0.667 [95% CI 0.564–0.763]), AISI (AUC = 0.662 [95% CI 0.553–0.768]), and NLR (AUC = 0.657 [95% CI 0.545–0.764]) had the best discriminative capacity, all with specificity >70%. The relation remained significant even after adjustments for confounding variables in multiple logistic regression. For microvascular complications, Monocyte count (AUC = 0.611 [95% CI 0.532–0.69]) and LMR (AUC = 0.608 [95% CI 0.521–0.695]) showed minimal but notable predictive value. *Conclusions:* SIRI, LMR, AISI, and NLR were significantly associated with mortality and demonstrated modest discriminative ability. These markers, accessible and cost-effective, may be useful tools for risk stratification in T2DM patients. Further validation in prospective cohorts is warranted.

## 1. Introduction

Type 2 diabetes mellitus (T2DM) represents a major global public health concern. Due to changes in the modern lifestyle, the prevalence of type 2 diabetes mellitus (T2DM) has increased alarmingly in recent decades. Twenty years ago, it was estimated that approximately 151 million adults were living with T2DM. By 2019, this number had risen to 463 million, and current projections suggest that by 2045, over 700 million people will be affected by this condition [1].

Cardiovascular disease (CVD) remains the leading cause of death among patients with T2DM. These include heart failure, stroke, myocardial infarction, and coronary artery disease, accounting for more than 50% of all deaths in this population [2].

Furthermore, over 80% of current T2DM cases are found in low- and middle-income countries, where healthcare systems often lack the necessary resources to manage chronic diseases properly. In contrast, in high-income countries, cardiovascular complications and all-cause mortality among T2DM patients have declined due to improved early detection, better access to medical care, and appropriate treatment strategies [3].

At the molecular level, chronic low-grade inflammation plays an important role in the development of insulin resistance and, consequently, in the pathogenesis of T2DM. Several cytokines and molecular pathways disrupt insulin signaling in the liver, skeletal muscle, and adipose tissue, contributing to metabolic dysfunction [4].

Systemic inflammation not only interferes with peripheral insulin action but also affects pancreatic beta-cell function. The chronic inflammatory environment marked by elevated levels of cytokines such as TNF-α, IL-6, and IL-1β, activates intracellular stress responses and oxidative damage, which impair insulin signaling and promote beta-cell apoptosis. This dual impact on both insulin sensitivity and insulin secretion promotes a pathological loop that accelerates metabolic deterioration and contributes to the progression of type 2 diabetes [5].

Based on this pathophysiological background, several blood-derived markers calculated from routine complete blood count, including the neutrophil-to-lymphocyte ratio (NLR), derived NLR (dNLR), monocyte-to-lymphocyte ratio (MLR/LMR), platelet-to-lymphocyte ratio (PLR), systemic immune-inflammation index (SII), systemic inflammation response index (SIRI), and aggregate index of systemic inflammation (AISI), have been proposed as surrogate markers of systemic inflammation and potential predictors of cardiovascular or all-cause mortality in patients with diabetes.

Beyond their prognostic relevance, these indices may also offer practical utility in preventive strategies. Their ability to identify high-risk individuals in early stages of the disease before clinical deterioration is an important feature that could support timely and targeted interventions. Integrating such markers into routine clinical workflows could therefore contribute not only to individualized risk stratification but also to reducing the incidence of diabetes-related complications.

One of the most comprehensive studies in this area, based on NHANES data from 2005 to 2018, confirmed that elevated levels of these indices are significantly associated with increased mortality risk [6].

SIRI and SII, in particular, have recently emerged as promising biomarkers with both diagnostic and prognostic value in cardio-metabolic conditions. Elevated levels have been significantly correlated with higher all-cause mortality over a 20-year follow-up period [7].

Concerning microvascular complications, both SII and SIRI have been identified as independent risk factors for diabetic retinopathy, chronic kidney disease, and diabetic neuropathy [8,9,10]. Furthermore, recent studies have demonstrated their association with macrovascular complications, including cardiovascular events, myocardial infarction, and stroke [11,12,13].

LMR is considered a prognostic marker in various chronic diseases. A higher LMR has been associated with lower mortality in patients with hypertension [14], although other studies have questioned its independent predictive value for cardiovascular death, emphasizing the superiority of NLR [15].

NLR, on the other hand, has consistently proven to be a simple, accessible, and reliable marker across numerous clinical studies and meta-analyses. Incorporating NLR into early risk assessment may help guide more rapid interventions to prevent cardiovascular decompensation and optimize diabetes management [16,17,18].

AISI has also been independently associated with increased all-cause mortality and higher rehospitalization rates for severe heart failure in patients undergoing PCI for acute coronary syndromes [19].

Interestingly, concerning lipid profiles, several studies have suggested a paradoxical association between low total cholesterol levels and increased mortality in specific populations, challenging the notion that “lower is always better” [20,21].

Although several hematological indices have been previously explored as predictors of cardiovascular events, few studies have conducted a comparative evaluation in hospitalized Eastern European diabetic populations. Moreover, there are very few studies that are longitudinal in nature; the majority are cross-sectional.

This study aimed to evaluate in a longitudinal manner the predictive value of several inflammatory indices derived from routine complete blood count, including NLR, dNLR, LMR, PLR, SII, SIRI, and AISI, in relation to the presence of vascular complications (both micro- and macrovascular) and cardiovascular mortality in patients with type 2 diabetes mellitus.

## 2. Materials and Methods

We conducted our study following the principles of the Declaration of Helsinki. It was approved by the Ethics Committee of the Bihor County Emergency Clinical Hospital (approval no. 34180, issued on 7 November 2024). All patients included in the study signed an informed consent form.

### 2.1. Study Design and Setting

This retrospective cohort study included 283 consecutive patients with type 2 diabetes who were admitted to the Bihor County Emergency Clinical Hospital between 1 January and 31 December 2022.

### 2.2. Participants

The study included patients diagnosed with type 2 diabetes mellitus, aged 18 years or older, with a complete set of available laboratory data. Exclusion criteria included patients with type 1 diabetes, LADA, those under 18 years of age, patients with autoimmune or hematological diseases, those undergoing immunomodulatory treatment, and patients with incomplete laboratory data. After applying these exclusion criteria, 237 patients were deemed eligible for analysis. All data were coded and anonymized before statistical processing to ensure the confidentiality of personal information.

### 2.3. Variables and Measurement

We followed the patients from the date of hospital admission (starting in January 2022) until death or the end of the study period in March 2025, whichever occurred first.

The median follow-up was 30 months (IQR 28-33).

The diagnosis of type 2 diabetes mellitus was established based on medical history and biological criteria, in accordance with the guidelines of the American Diabetes Association (ADA, 2024) and current European recommendations [22].

Hypertension, dyslipidemia, and other cardiovascular comorbidities were recorded according to standard clinical definitions, as documented in each patient’s medical history.

For each patient, the following general data were collected: age, sex, place of residence (urban/rural), smoking status, duration of diabetes, as well as anthropometric parameters such as weight, height, and body mass index (BMI). No follow-up data on changes in place of residence were available. Classification was based on the patients’ declared address at the time of hospital admission.

The presence of chronic complications was assessed based on existing medical records. It included macrovascular complications (myocardial infarction, stroke, peripheral arterial disease) and microvascular complications (diabetic retinopathy, diabetic polyneuropathy, chronic kidney disease).

All laboratory analyses were performed using standardized methods in the central laboratory of the Bihor County Emergency Clinical Hospital. Blood samples were collected under fasting conditions (à jeun) during routine clinical evaluations. The biochemical parameters analyzed included complete blood count (CBC); fasting glucose; glycated hemoglobin (HbA1c); total cholesterol, LDL cholesterol, HDL cholesterol, triglycerides; uric acid, creatinine, urea—used to calculate the Estimated Glomerular Filtration Rate (eGFR), C-reactive protein (CRP), and Erythrocyte Sedimentation Rate (ESR).

From the CBC, the absolute values of neutrophils, lymphocytes, monocytes, and platelets were extracted and used to calculate the following derived inflammatory indices: NLR (neutrophil-to-lymphocyte ratio) = neutrophils/lymphocytes; PLR (platelet-to-lymphocyte ratio) = platelets/lymphocytes; MLR (monocyte-to-lymphocyte ratio) = monocytes/lymphocytes; dNLR (derived neutrophil-to-lymphocyte ratio) = neutrophils/(white blood cells—neutrophils); SII (systemic immune-inflammation index) = (neutrophils × platelets)/lymphocytes; SIRI (systemic inflammation response index) = (neutrophils × monocytes)/lymphocytes; AISI (aggregate index of systemic inflammation) = (neutrophils × monocytes × platelets)/lymphocytes.

All biological parameters included in the analysis were obtained at the time of patient inclusion in the study, using the most recent values available in the electronic medical record.

The outcome of interest was death and microvascular or macrovascular complications; the exposure variables included hematological markers.

### 2.4. Statistical Analysis

Qualitative variables were summarized as frequencies and percentages, while non-normally distributed quantitative variables were reported as medians with interquartile ranges (IQR). Comparisons between two independent groups were conducted using the chi-squared test or Fisher’s exact test for categorical variables and the Wilcoxon rank-sum test for continuous variables with non-normal distributions. The diagnostic performance of hematological markers was evaluated using receiver operating characteristic (ROC) curves, with the area under the curve (AUC) and corresponding 95% confidence intervals (CI) reported. Optimal cut-off values for each marker were determined by maximizing the Youden index (sensitivity + specificity − 1). Sensitivity and specificity were subsequently calculated for each selected cut-off. Multivariate logistic regression models predicting death based on blood cell count indices as binary variables using their cut-offs were intended to be adjusted for age, diabetes duration, body mass index, number of comorbidities, and number of treatments for diabetes. Since only 30 deaths were observed, the maximum number of independent variables to prevent overfitting was 3, using the rule of 10 participants per variable, in the smallest class. To be able to include the relevant information from all the intended adjustment variables, a principal component analysis was performed, and the first two components were used in the multivariate regression as adjustments. To ensure the effect of HbA1c was taken into account, a follow-up model including this variable was also fitted for each variable of interest. For all models, the multicollinearity was checked using the variance inflation factor, the goodness-of-fit was checked with the Hosmer and Lemeshow test, while the assumption of linearity to the logit for the continuous independent variables was checked with smooth terms in a general additive model. All statistical tests were two-tailed, with a significance threshold set at *p* < 0.05. Analyses were performed using R version 4.3.2 (R Foundation for Statistical Computing, Vienna, Austria).

During the preparation of this manuscript, the authors used ChatGPT (OpenAI, 4.0, June 2024) as a brainstorming tool to generate suggestions for perspectives to include in the Discussion section. Also, it was used to improve the scientific writing of the manuscript. The authors have reviewed and edited the output and take full responsibility for the content of this publication.

## 3. Results

The final study cohort included 237 patients diagnosed with type 2 diabetes mellitus (T2DM), of whom 30 (12.66%) died during the analyzed period. There was no statistically significant age difference between deceased and surviving patients. Additionally, no significant differences were observed between the two groups regarding sex, place of residence, smoking status, or diabetes duration.

The analysis of diabetes-associated comorbidities and complications revealed several relevant differences. Diabetic retinopathy was significantly more prevalent in the deceased group (*p* = 0.027). A history of stroke was significantly more common among deceased patients (*p* = 0.01). Overall, macrovascular complications were more frequent in deceased patients, with a borderline statistical significance (*p* = 0.05). The main clinical characteristics are summarized in Table 1.

Laboratory comparisons are detailed in Table 2. Hematological inflammatory markers showed significantly higher values among deceased patients for the following parameters: White Blood Cell count (WBC, *p* = 0.03), neutrophils (NEU, *p* = 0.013), monocytes (MON, *p* = 0.013), neutrophil-to-lymphocyte ratio (NLR, *p* = 0.006), derived NLR (dNLR, *p* = 0.021), lymphocyte-to-monocyte ratio (LMR, *p* = 0.003), systemic immune-inflammation index (SII, *p* = 0.034), systemic inflammation response index (SIRI, *p* = 0.001), and aggregate index of systemic inflammation (AISI, *p* = 0.004), compared to survivors.

All results are presented as median and interquartile range; WBC, White Blood Cell count; NEU, Neutrophil count; LYM, Lymphocyte count; MONO, Monocyte count; RBC, Red Blood Cell count; HGB, Hemoglobin; MCV, Mean Corpuscular Volume; PLT, Platelet count; NLR, neutrophil-to-lymphocyte ratio; dNLR, derived neutrophil-to-lymphocyte ratio; PLR, platelet-to-lymphocyte ratio; LMR, lymphocyte-to-monocyte ratio; SII, systemic immune-inflammation index; SIRI, systemic inflammation response index; AISI, aggregate index of systemic inflammation; TRG, Triglycerides; HDL-chol, High-Density Lipoprotein Cholesterol; GOT, Aspartate Aminotransferase (AST); GPT, Alanine Aminotransferase (ALT); Urea, Serum Urea; Creatinine, Serum Creatinine; ESR, Erythrocyte Sedimentation Rate; eGFR = CKD-EPI, Estimated Glomerular Filtration Rate calculated by the CKD-EPI formula; TyG Index, Triglyceride-Glucose Index.

In addition, total cholesterol levels were significantly lower in deceased patients compared to survivors (*p* = 0.047).

**Figure 1:**
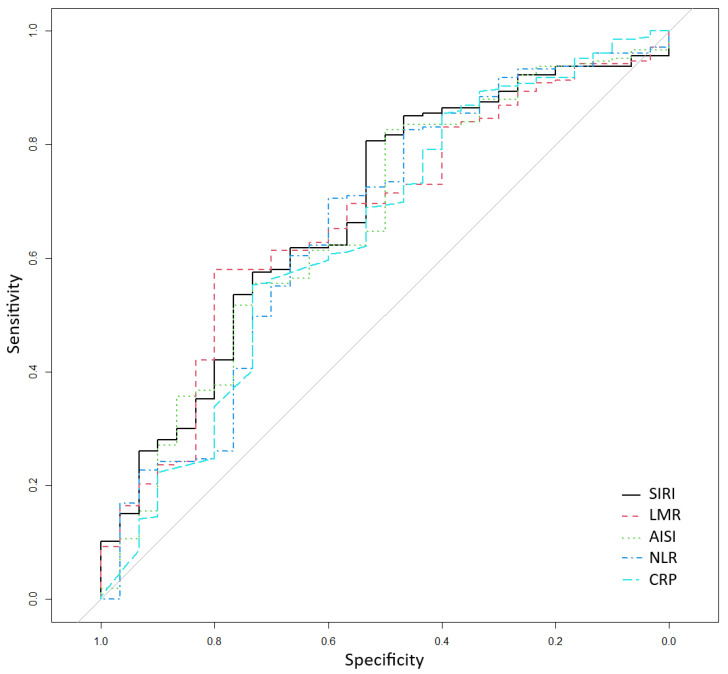
Receiver operating characteristic for classifying death according to systemic inflammation response index (SIRI), lymphocyte-to-monocyte ratio (LMR), aggregate index of systemic inflammation (AISI), neutrophil-to-lymphocyte ratio (NLR), and CRP (C-reactive protein).

The ROC curve analysis, presented in Table 3 and Figure 1, was used to assess the performance of inflammatory markers in classifying the risk of death. The most predictive indices were SIRI (AUC = 0.680), LMR (AUC = 0.667), AISI (AUC = 0.662), and NLR (AUC = 0.657), all showing specificity values above 70% and AUCs close to or slightly above 0.65. Sensitivity values ranged between 50% and 60% across the main indices. To further check the discriminatory ability of these indices using their cut-offs, we included them in multivariate logistic regression models predicting death, adjusted for age, diabetes duration, body mass index, number of comorbidities, and number of treatments for diabetes. All four indices remained statistically significant associated with death, with higher values of SIRI, AISI, and NLR increasing the odds of death, while higher values of LMR decreasing the odds of death (Table 4). Finally, for each of the previous models, we further adjusted for the glycemic control (HbA1c < 6.5%), and the results remain very similar to the initial multivariate models, supporting the robustness of the indices, even after controlling for the essential factor of glycemic control.

The ROC analysis also showed that hematological inflammatory indices performed slightly better in predicting microvascular complications compared to macrovascular ones.

In the context of microvascular complications (Table 5), the highest AUC values were observed for Monocyte count (AUC = 0.611) and LMR (AUC = 0.608). Although these values do not meet the criteria for acceptable discriminatory power, they were higher than those of the other markers, suggesting a weak but possible association between systemic inflammation and microvascular damage in patients with T2DM.

Conversely, for macrovascular complications (Table 6), all markers demonstrated poor predictive performance, with none exceeding an AUC of 0.60. The best-performing parameters were RBC and ESR (both AUC = 0.583), and LMR (AUC = 0.573). Classical inflammatory indices such as NLR, SIRI, AISI, dNLR, and SII showed even lower AUC values, further supporting their limited utility in detecting macrovascular involvement within this cohort.

## 4. Discussion

This study evaluated the predictive value of several inflammatory indices derived from routine complete blood count (including NLR, dNLR, LMR, PLR, SII, SIRI, and AISI) concerning vascular complications and cardiovascular mortality in patients with type 2 diabetes mellitus (T2DM). The results revealed significant differences in these indices between deceased and surviving patients, suggesting an association between systemic inflammation and cardiovascular prognosis. Furthermore, some indices—such as SIRI, LMR, and AISI—demonstrated a moderate discriminative capacity for predicting mortality, highlighting their potential as accessible and reproducible biomarkers for risk stratification in this patient population.

Although no statistically significant differences were observed regarding sex, the median age was slightly higher among deceased patients (70.5 vs. 67 years), and the median duration of diabetes was longer (13.5 vs. 10 years), which may reflect a cumulative risk associated with disease progression and aging.

Variables such as sex, place of residence (urban or rural), and smoking status did not show significant differences between groups. However, a slightly higher proportion of deceased patients came from urban areas. This observation aligns with data from the International Diabetes Federation (IDF), which indicates a higher prevalence of T2DM in urban areas (12.1%) compared to rural regions, likely due to sedentary lifestyles and poor dietary habits [23].

Regarding body mass index (BMI), median values were similar between groups, suggesting that inflammatory factors may contribute to prognosis independently of body weight. Moreover, in the multivariate logistic regression model, we controlled for the effect of BMI to isolate the independent effect of the inflammatory markers. These findings support the notion that systemic inflammation could represent a common and relevant mechanism of disease progression, regardless of demographic or anthropometric characteristics.

Our analysis revealed significantly higher levels of leukocytes, neutrophils, and monocytes in deceased patients. These findings are consistent with the NHANES 2023 study [7], which demonstrated a correlation between leukocytosis and cardiovascular mortality in diabetic populations.

Neutrophils, as key components of the natural immune response, contribute to atherosclerotic plaque instability [24], while monocytes play a crucial role in the formation of proinflammatory macrophages that exacerbate endothelial dysfunction. In a study from 2022 [25], monocytosis was significantly connected with the severity of diabetic retinopathy—an association also observed in our cohort. In addition, recent findings from a 2023 retrospective cohort study [26] demonstrated that an elevated monocyte-to-lymphocyte ratio (MLR) independently predicted 90-day all-cause mortality in patients with type 2 diabetes and chronic kidney disease, further supporting the inflammatory contribution of monocytes to poor short-term prognosis in high-risk diabetic populations.

Among the derived inflammatory indices analyzed, SIRI, LMR, AISI, and NLR demonstrated the best predictive performance for mortality in our cohort. These findings are supported by a large nationwide cohort study based on NHANES 2005–2018 data, which evaluated six inflammatory biomarkers (NLR, dNLR, PLR, SII, SIRI, and AISI) and identified a significant association between elevated values and the risk of all-cause mortality in patients with type 2 diabetes mellitus. Among them, SIRI showed the strongest prognostic performance, remaining an independent predictor even after adjusting for factors such as age, sex, ethnicity, comorbidities, and antidiabetic treatments [6]. Similarly, other studies [19,27], have identified SII and AISI as significant predictors of major cardiac events in diabetic patients post-PCI. Moreover, a large population-based cohort study [28] confirmed that elevated SII values are independently associated with both all-cause and cardiovascular mortality in patients with type 2 diabetes, further reinforcing its role as a valuable inflammatory marker for long-term risk stratification.

NLR (AUC = 0.657) also proved to be a moderately predictive marker in our study, consistent with a recent meta-analysis by Sun et al. in 2023 [16], which confirms its prognostic role in T2DM. Furthermore, this finding is supported by a recent systematic review and meta-analysis published in 2025 [29], which emphasized the strong and consistent association between elevated NLR levels and the risk of diabetic nephropathy, further highlighting its utility as an accessible and non-invasive indicator of microvascular damage in type 2 diabetes. Additional evidence from another meta-analysis [30] also confirmed that NLR is significantly elevated in patients with both diabetic retinopathy and nephropathy, reinforcing its relevance as a marker of microvascular complications across multiple target organs.

On the other hand, SII and PLR, although promising in other studies [7,31], did not show convincing predictive value in our cohort.

It is important to note that various antidiabetic and cardiometabolic therapies, such as metformin, statins, or ACE inhibitors, are known to exert anti-inflammatory effects and may potentially influence the circulating levels of hematologic inflammatory markers. Therefore, elevated SIRI, NLR, or AISI values may not always reflect uncontrolled inflammation but could also indicate a differential therapeutic response. Although our analysis adjusted for the overall number of treatments, we were not able to assess the interaction between specific therapies and marker performance due to the limited number of events in the cohort. This represents an important area for future research, as understanding how inflammatory indices behave across different therapeutic backgrounds would be essential for their personalized interpretation and reliable integration into clinical decision-making.

A distinct observation in our analysis was the association between low total cholesterol and increased mortality, known in the literature as the “cholesterol paradox”, possibly explained by chronic low-grade inflammation or underlying biological frailty [21].

Similarly, a study by Yun Gi Kim et al. reported a U-shaped relationship between total cholesterol and all-cause mortality, suggesting that both markedly low and high cholesterol levels may be associated with adverse outcomes. These findings emphasize the importance of interpreting lipid profiles with caution, particularly in vulnerable populations such as patients with advanced T2DM and persistent systemic inflammation [32].

Several mechanisms have been proposed to explain this paradox. In the context of inflammation, hepatic cholesterol synthesis may be downregulated via cytokine-mediated pathways, most notably through IL-6-induced STAT3 activation, resulting in reduced circulating cholesterol levels. Rather than indicating cardiovascular protection, such reductions may reflect impaired metabolic reserve and resilience. Furthermore, low cholesterol concentrations are frequently observed in states of malnutrition, sarcopenia, or chronic catabolism, which are common among elderly or frail individuals. Since cholesterol is vital for cellular membrane integrity, steroid hormone production, and immune function, insufficient levels could impair tissue repair and overall survival capacity [20].

### 4.1. Strengths

One of the key strengths of this study is the simultaneous and comparative evaluation of multiple hematologic inflammatory indices, including NLR, PLR, dNLR, LMR, SII, SIRI, and AISI. This approach not only allowed identification of individual markers with prognostic value but also facilitated the establishment of a comparative hierarchy based on statistical performance (AUC), contributing to the selection of the most clinically relevant indices. The majority of current studies assessing these inflammatory indices are cross-sectional, while our research is longitudinal, providing evidence for the prognostic role of these indices.

Additionally, all evaluated markers can be calculated from routine blood tests, making their implementation in everyday clinical practice feasible, fast, and low-cost. This is particularly valuable in healthcare systems with limited resources, where advanced imaging or molecular testing is not always available.

### 4.2. Limitations

This study has several limitations that must be considered. First, its retrospective and single-center design may introduce selection bias and limit the generalizability of the results. Furthermore, derived indices such as NLR, SIRI, or AISI can be influenced by intercurrent conditions (e.g., infections, hematologic disorders) that could not be entirely ruled out retrospectively. Being an observational study, causation cannot be sustained. The lack of a longer longitudinal follow-up prevented us from evaluating the dynamic behavior of these markers over time. Although several indices showed statistically significant associations with mortality, their discriminative performance was modest, with AUC values between 0.65 and 0.68 and sensitivity generally below 60%. These values suggest a limited ability to accurately classify individual risk in a clinical setting. Therefore, while such markers may support broader epidemiological or risk-stratification efforts, they are not reliable as standalone predictors. Their utility may increase when incorporated into multifactorial risk assessment models or used alongside clinical parameters. Further prospective validation is necessary to determine their true value in guiding patient management.

### 4.3. Future Perspectives

Our findings support the need for future longitudinal, prospective, multicenter studies to validate the prognostic utility of inflammatory indices in assessing cardiovascular risk among patients with T2DM. Monitoring these markers dynamically concerning clinical evolution and treatment response could provide additional insights into their prognostic relevance. Furthermore, incorporating them into multifactorial risk models may enhance the accuracy of clinical decision-making.

## 5. Conclusions

Inflammatory indices such as SIRI, LMR, AISI, and NLR showed moderate predictive value for mortality in patients with type 2 diabetes, supporting their role as accessible tools for risk stratification. Their association with systemic inflammation and disease progression highlights the need for further validation in prospective, multicenter studies.

## Figures and Tables

**Table 1:** Patients’ characteristics.

Characteristics	Death (*n* = 30)	Alive (*n* = 207)	*p*-Value
Age, median (IQR)	70.5 (65–76.5)	67 (60.5–73)	0.06
Sex (F), *n* (%)	13 (43.33)	118 (57)	0.159
Place of residence (Urban/Rural) (R), *n* (%)	15 (50)	102 (49.51)	0.96
Smoking status, *n* (%)	3 (10)	29 (14.01)	0.776
Diabetes duration, median (IQR)	13.5 (9–16)	10 (4–15)	0.086
Body Mass Index (BMI), median (IQR)	28.37 (23.63–33.55)	29.74 (25.87–32.88)	0.351
Systolic Blood Pressure, median (IQR)	142.5 (131–158.75)	140 (130.5–155)	0.965
Essential Arterial Hypertension, *n* (%)	26 (86.67)	185 (89.37)	0.753
Chronic Heart Failure, *n* (%)	12 (40)	55 (26.57)	0.127
Chronic Ischemic Heart Disease, n (%)	14 (46.67)	93 (44.93)	0.858
Left Ventricular Failure, *n* (%)	0 (0)	23 (11.11)	0.09
Dyslipidemia, *n* (%)	11 (36.67)	88 (42.51)	0.544
Weight status, *n* (%)			0.369
Obesity	13 (43.33%)	92 (44.44%)	
Overweight	0 (0%)	16 (7.73%)	
Normal weight	17 (56.67%)	98 (47.34%)	
Underweight	0 (0%)	1 (0.48%)	
Diabetic Polyneuropathy, *n* (%)	9 (30)	95 (45.89)	0.101
Diabetic Retinopathy, *n* (%)	1 (3.33)	41 (19.81)	0.027
Chronic Kidney disease, *n* (%)	10 (33.33)	86 (41.55)	0.392
History of Myocardial Infarction, *n* (%)	0 (0)	12 (5.8)	0.372
History of Stroke, *n* (%)	8 (26.67)	19 (9.18)	0.01
Peripheral Arterial Disease, *n* (%)	6 (20)	27 (13.04)	0.394
Hepatic Steatosis, *n* (%)	13 (43.33)	99 (47.83)	0.645
Thyroid disorders, *n* (%)	3 (10)	20 (9.66)	1
Pancreatitis, *n* (%)	0 (0)	2 (0.97)	1
Psychiatric disorders, *n* (%)	2 (6.67)	17 (8.21)	1
Dermatological disorders, *n* (%)	0 (0)	1 (0.48)	1
Pulmonary disorders, *n* (%)	10 (33.33)	38 (18.36)	0.056
Oncological history, *n* (%)	5 (16.67)	16 (7.73)	0.158
Microvascular complications, *n* (%)	17 (56.67)	152 (73.43)	0.058
Macrovascular complications, *n* (%)	13 (43.33)	54 (26.09)	0.05

**Table 2:** Hematological inflammatory indexes.

Characteristics	Death (*n* = 30)	Alive (*n* = 207)	*p*-Value
Fasting blood sugar (mg/dL)	213.5 (125–329)	176 (132–288.5)	0.883
WBC (10^3^/uL)	9.93 (7.19–14.23)	8.15 (6.72–10.4)	0.03
NEU (10^3^/uL)	6.58 (4.92–11.8)	5.27 (4.2–6.99)	0.013
LYM (10^3^/uL)	1.6 (1.08–2.08)	1.88 (1.27–2.39)	0.171
MONO (10^3^/uL)	0.74 (0.58–0.96)	0.6 (0.5–0.76)	0.013
RBC (10^6^/uL)	4.41 (3.69–4.69)	4.5 (4.11–4.91)	0.059
HGB(g/dL)	13.1 (11.15–14.3)	13.5 (12.3–14.75)	0.09
MCV (fL)	92.95 (88.22–99.15)	92.5 (89.15–95.65)	0.38
PLT (10^3^/uL)	246 (206.75–310.75)	244 (191–300)	0.564
NLR	4.45 (2.62–10.35)	3 (1.99–4.55)	0.006
dNLR	2.62 (1.66–4.54)	1.99 (1.44–2.91)	0.021
PLR	147.11 (105.73–186.25)	132.28 (96.75–189.34)	0.534
LMR	2.2 (1.35–2.83)	3.15 (2.04–4.17)	0.003
SII	1089.02 (568.09–2410.62)	686.98 (429.64–1142.45)	0.034
SIRI	3.84 (1.89–8.33)	1.71 (1.1–2.86)	0.001
AISI	799.05 (448.05–2291.47)	419.19 (229.65–789.86)	0.004
Total cholesterol (mg/dL)	145 (111–177)	163 (135–190)	0.047
TRG(mg/dL)	120 (100–165)	127 (94–170)	0.951
HDL-col (mg/dL)	41 (33–44)	40 (34–48)	0.418
GOT (U/L)	23.5 (17.25–31.75)	22 (17–28)	0.464
GPT (U/L)	20.5 (17–28.25)	22 (16–32.5)	0.675
Urea (mg/dL)	16 (10.25–28.25)	18 (14–26)	0.282
Creatinine (mg/dL)	0.98 (0.73–1.49)	0.92 (0.77–1.25)	0.755
VSH (mm/h)	22.5 (11–42.5)	16 (10–28)	0.253
RFG = CKD−EPI (mL/min/1.73 m^2^)	67.12 (41.21–92.45)	70.47 (48.78–90.48)	0.649
TyG Index	−0.2 (−0.47–0.01)	−0.2 (−0.42–0.04)	0.837

**Table 3:** Receiver operating characteristic analysis for death outcome classification.

Variable	AUC (95% CI)	Se	Sp	Cut-Off
SIRI	0.68 (0.576–0.779)	53.33	80.68	3.596992
LMR	0.667 (0.564–0.763)	57.97	80	2.842809
AISI	0.662 (0.553–0.768)	50	82.61	978.8176
NLR	0.657 (0.545–0.764)	60	70.53	4.23301
CRP	0.642 (0.532–0.756)	73.33	55.34	4.4
MONO	0.641 (0.526–0.745)	66.67	59.9	0.644
NEU	0.64 (0.526–0.746)	43.33	78.74	7.46
dNLR	0.63 (0.516–0.743)	46.67	78.26	3.047619
WBC	0.623 (0.511–0.735)	66.67	56.52	8.6
SII	0.62 (0.492–0.732)	50	74.88	1134
RBC	0.607 (0.493–0.718)	87.92	36.67	3.72
LYM	0.577 (0.46–0.682)	3.33	100	5.21
ESR	0.565 (0.438–0.69)	43.33	81.86	32
MCV	0.55 (0.422–0.675)	30	89.37	98.6
PLR	0.535 (0.414–0.647)	53.33	58.45	144.6667
PLT	0.533 (0.407–0.651)	73.33	40.58	217
TyG Index	0.512 (0.389–0.63)	65.37	48.28	−0.28435

AUC, area under the curve; CI, confidence interval; Se, sensitivity; Sp, specificity; SIRI, systemic inflammation response index; LMR, lymphocyte-to-monocyte ratio; AISI, aggregate index of systemic inflammation; NLR, neutrophil-to-lymphocyte ratio; CRP, C-reactive protein (mg/L); MONO, Monocyte count (10^3^/μL); NEU, Neutrophil count (10^3^/μL); dNLR, derived neutrophil-to-lymphocyte ratio; WBC, White Blood Cell count (10^3^/μL); SII, systemic immune-inflammation index; RBC, Red Blood Cell count (10^6^/μL); LYM, Lymphocyte count (10^3^/μL); ESR, Erythrocyte Sedimentation Rate (mm/h); MCV, Mean Corpuscular Volume (fL); PLR, platelet-to-lymphocyte ratio; PLT, Platelet count (10^3^/μL); TyG Index, Triglyceride-Glucose Index.

**Table 4:** Multivariate logistic regression models predicting death based on blood cell count indices, adjusted for age, diabetes duration, body mass index, number of comorbidities, and number of treatments for diabetes; a second model for each variable of interest includes a further adjustment for glycemic control (HbA1c < 6.5%).

	Model 1			Model 1 + HbA1c < 6.5 (%)		
Characteristic	OR Adjusted	(95% CI)	*p*	OR Adjusted	(95% CI)	*p*
SIRI ≥ 3.60	5.03	(2.21–11.64)	<0.001	5.35	(2.30–12.75)	<0.001
LMR ≥ 2.84	0.25	(0.09–0.6)	0.003	0.25	(0.09–0.61)	<0.001
AISI ≥ 978.82	5.08	(2.2–11.89)	<0.001	5.28	(2.22–12.80)	<0.001
NLR ≥4.23	3.38	(1.52–7.8)	0.003	3.29	(1.45–7.68)	0.005

SIRI, systemic inflammation response index; LMR, lymphocyte-to-monocyte ratio; AISI, aggregate index of systemic inflammation; NLR, neutrophil-to-lymphocyte ratio; OR, odds ratio; CI, confidence interval.

**Table 5:** Microvascular complications.

Variable	AUC (95% CI)
MONO	0.611 (0.532–0.69)
LMR	0.608 (0.521–0.695)
SIRI	0.586 (0.504–0.67)
AISI	0.578 (0.496–0.662)
PLR	0.56 (0.475–0.646)
LYM	0.553 (0.464–0.639)
NLR	0.54 (0.459–0.619)
SII	0.54 (0.457–0.617)
RBC	0.537 (0.453–0.616)
CRP	0.525 (0.446–0.608)
NEU	0.523 (0.436–0.603)
TyG Index	0.519 (0.434–0.6)
dNLR	0.507 (0.423–0.584)
MCV	0.503 (0.429–0.579)
ESR	0.502 (0.414–0.59)
PLT	0.499 (0.411–0.584)
WBC	0.482 (0.399–0.566)

MONO, Monocyte count (10^3^/μL); LMR, lymphocyte-to-monocyte ratio; SIRI, systemic inflammation response index; AISI, aggregate index of systemic inflammation; PLR, platelet-to-lymphocyte ratio; LYM, Lymphocyte count (10^3^/μL); NLR, neutrophil-to-lymphocyte ratio; SII, systemic immune-inflammation index; RBC, Red Blood Cell count (10^6^/μL); CRP, C-reactive protein (mg/L); NEU, Neutrophil count (10^3^/μL); TyG Index, Triglyceride-Glucose Index; dNLR, derived neutrophil-to-lymphocyte ratio; MCV, Mean Corpuscular Volume (fL); ESR, Erythrocyte Sedimentation Rate (mm/h); PLT, Platelet count (10^3^/μL); WBC, White Blood Cell count (10^3^/μL).

**Table 6:** Macrovascular complications.

Variable	AUC (95% CI)
RBC	0.583 (0.504–0.665)
VSH	0.583 (0.5–0.66)
LMR	0.573 (0.491–0.656)
MONO	0.561 (0.478–0.643)
LYM	0.556 (0.471–0.642)
WBC	0.542 (0.465–0.624)
NEU	0.537 (0.455–0.624)
SIRI	0.537 (0.454–0.621)
CRP	0.527 (0.446–0.604)
MCV	0.527 (0.445–0.605)
PLR	0.52 (0.435–0.605)
TyG Index	0.517 (0.436–0.595)
NLR	0.516 (0.427–0.602)
PLT	0.504 (0.419–0.583)
SII	0.499 (0.416–0.586)
dNLR	0.493 (0.408–0.577)
AISI	0.478 (0.399–0.563)

RBC, Red Blood Cell count (10^6^/μL); ESR, Erythrocyte Sedimentation Rate (mm/h); LMR, lymphocyte-to-monocyte ratio; MONO, Monocyte count (10^3^/μL); LYM, Lymphocyte count (10^3^/μL); WBC, White Blood Cell count (10^3^/μL); NEU, Neutrophil count (10^3^/μL); SIRI, systemic inflammation response index; CRP, C-reactive protein (mg/L); MCV, Mean Corpuscular Volume (fL); PLR, platelet-to-lymphocyte ratio; TyG Index, Triglyceride-Glucose Index; NLR, neutrophil-to-lymphocyte ratio; PLT, Platelet count (10^3^/μL); SII, systemic immune-inflammation index; dNLR, derived neutrophil-to-lymphocyte ratio; AISI, aggregate index of systemic inflammation.

## Data Availability

The raw data supporting the conclusions of this article will be made available by the authors on request.

## Abbreviations

The following abbreviations are used in this manuscript:

Abbreviation	Explanation
WBC	White Blood Cell count
NEU	Neutrophil count
LYM	Lymphocyte count
MONO	Monocyte count
RBC	Red Blood Cell count
HGB	Hemoglobin
MCV	Mean Corpuscular Volume
PLT	Platelet count
NLR	Neutrophil-to-Lymphocyte Ratio
dNLR	Derived Neutrophil-to-Lymphocyte Ratio
PLR	Platelet-to-Lymphocyte Ratio
LMR	Lymphocyte-to-Monocyte Ratio
SII	Systemic Immune-Inflammation Index
SIRI	Systemic Inflammation Response Index
AISI	Aggregate Index of Systemic Inflammation
TRG	Triglycerides
HDL-chol	High-Density Lipoprotein Cholesterol
GOT	Aspartate Aminotransferase (AST)
GPT	Alanine Aminotransferase (ALT)
Urea	Serum Urea
Creatinine	Serum Creatinine
ESR	Erythrocyte Sedimentation Rate
eGFR = CKD-EPI	Estimated Glomerular Filtration Rate calculated by the CKD-EPI formula
TyG Index	Triglyceride-Glucose Index
CI	Confidence Interval
IQR	Interquartile Range
OR	Odds Ratio
ROC	Receiver Operating Characteristic
AUC	Area Under the ROC Curve
Se	Sensitivity
Sp	Specificity
BMI	Body Mass Index
T2DM	Type 2 Diabetes Mellitus
CVD	Cardiovascular Disease
CRP	C-reactive protein
